# Supplementary material for: PatientProfiler: building patient-specific signaling models from proteogenomic data
Source: Mol Syst Biol. 2025 Oct 10;21(12):1845–65. doi: 10.1038/s44320-025-00160-y (PMC12672659; doi:10.1038/s44320-025-00160-y)
Supplement: Supplementary file 1 — Appendix [file 44320_2025_160_MOESM1_ESM.pdf]

Appendix on
PatientProfiler: A network-based approach to personalized medicine

Table of contents

Figure	Page number
Appendix Figure S1	2
Appendix Figure S2	3
Appendix Figure S3	4
Appendix Figure S4	5
Appendix Figure S5	6
Appendix Figure S6	7
Appendix Figure S7	8
Appendix Figure S8	9
Appendix Figure S9	10
Appendix Figure S10	11
Appendix Figure S11	12
Appendix Figure S12	13
Appendix Figure S13	14
Appendix Figure S14	15
Appendix Figure S15	16
Appendix Figure S16	17
Appendix Figure S17	18
Appendix Figure S18	19
Appendix Figure S19	20
Appendix Figure S20	21
Appendix Figure S21	22
Appendix Figure S22	23

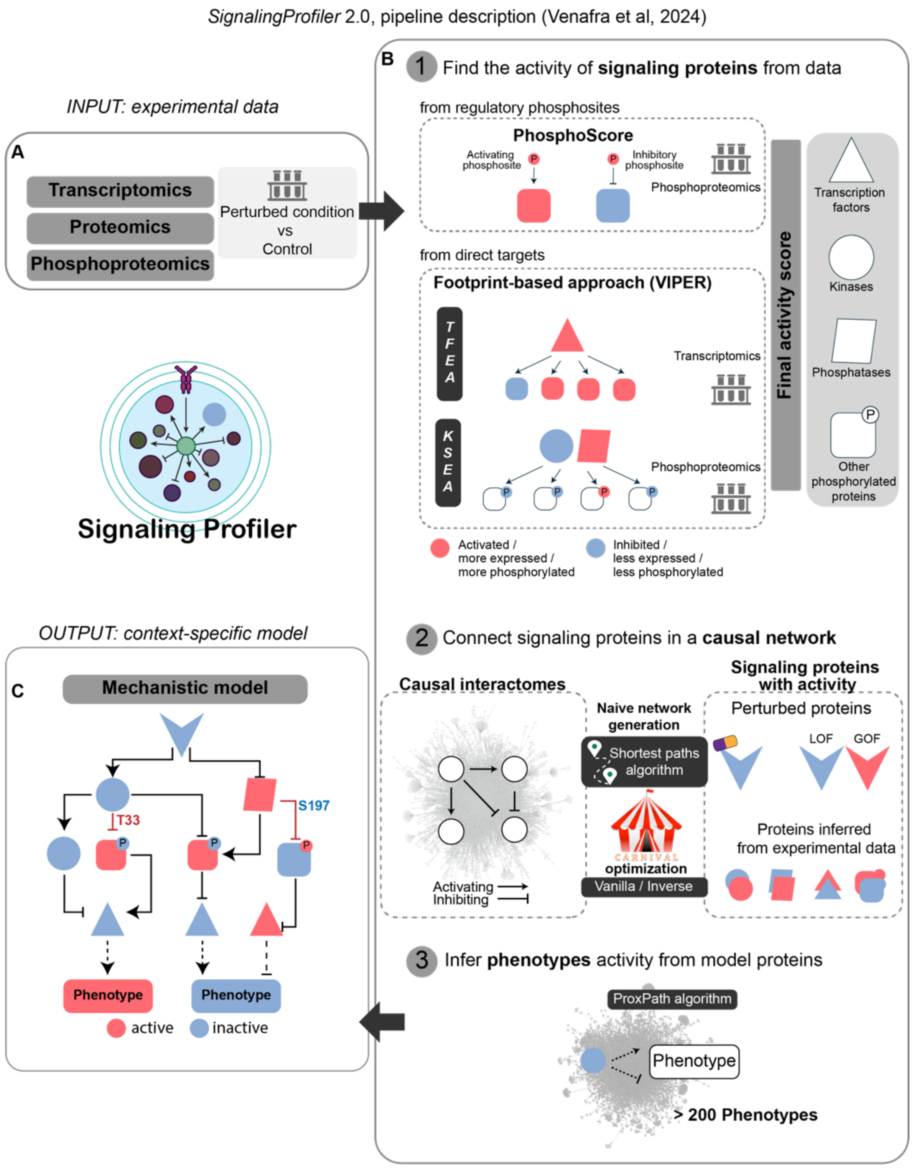

Appendix Figure S1 ***SignalingProfiler*** **2.0, detailed description** (Venafra *et al*, 2024)**.** *SignalingProfiler* 2.0 integrates multi-omics experimental data (A) with prior-knowledge information (B), to derive context-specific mechanistic models (C). **A)** *SignalingProfiler* 2.0 accept as input experimentally-collected transcriptomics and (phospho)proteomics. **B)** In Step 1 it derives the activity of signaling proteins using the footprint-based approach and the PhosphoScore algorithms. In the footprint-based approach, *SignalingProfiler* 2.0 leverages the VIPER inference (Alvarez *et al*, 2016) to derive the activity of transcription factors, kinases, and phosphatases based on the abundance of their target transcripts or phosphopeptides in the transcriptomics and phosphoproteomics, respectively. The PhosphoScore algorithm combines the modulation of phosphosites in phosphoproteomics data with their impact on protein activity/stability as annotated in PhosphoSitePlus (Hornbeck *et al*, 2015) and SIGNOR (Lo Surdo *et al*, 2023), to estimate the activity of phosphorylated proteins. In Step 2 perturbed proteins (e.g., targets of a treatment or mutated genes in a disease) are connected to the inferred proteins (Step1) using a prior knowledge network (PKN). The so-generated network (naïve network) is optimized with the CARNIVAL optimization strategy (Liu *et al*, 2019) to retain only the sign-coherent interactions between proteins. Step 3 The context-specific network is further connected to cellular phenotypes leveraging the ProxPath algorithm (Iannuccelli *et al*, 2023). **C)** The output of *SignalingProfiler* 2.0 is a context-specific mechanistic model that illustrate the remodeling of the signaling at the phosphorylation-resolution level.

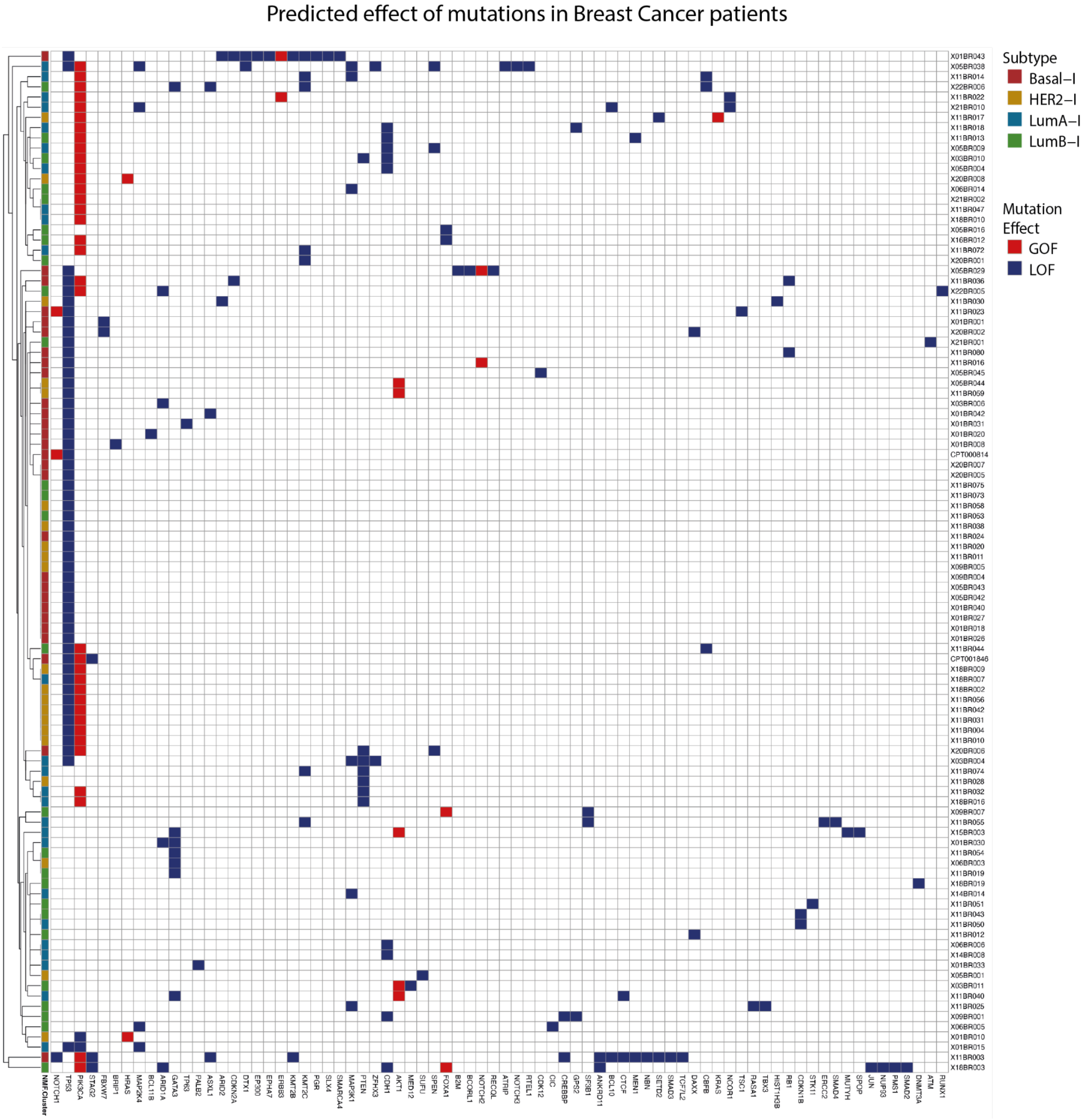

Appendix Figure S2 Clustering based on the mutational profile of breast Cancer Patients. Breast cancer genomic data was retrieved from the CPTAC portal. Briefly, we exploited OncoKB (https://www.oncokb.org/)(Chakravarty *et al*, 2017) to estimate the functional impact of mutations and to derive their relative impact on protein activity: loss-of-function (LOF - in blue) and gain-of-function (GOF - in red) mutations were associated to inactivation or activation of the target protein, respectively.

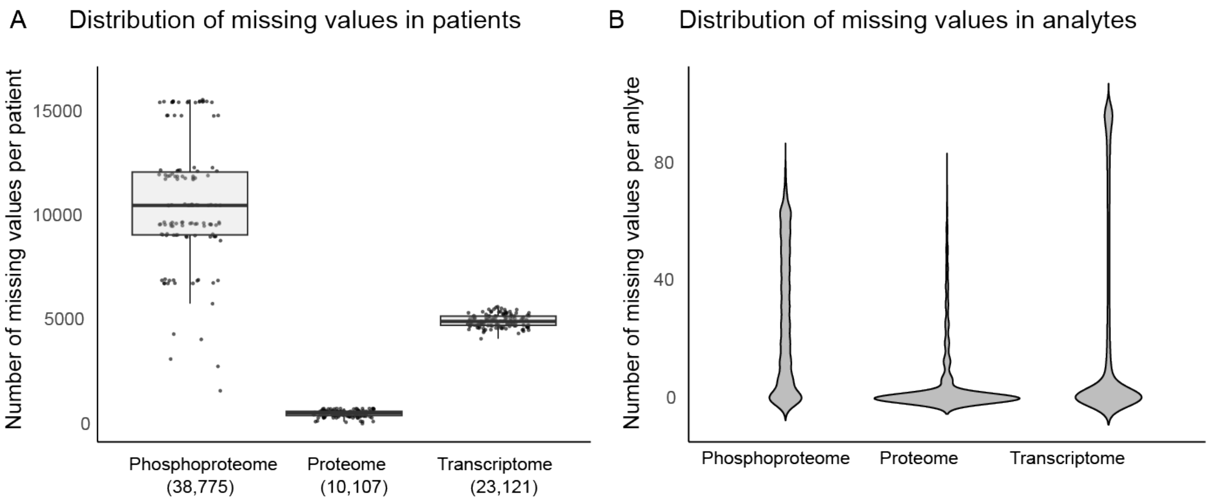

Appendix Figure S3. Missing values in CPTAC datasets. A) Distribution of number of missing values within the same sample (patient), in parenthesis the total number of analytes in each omic level. **B)** Percentage of missing values within the same analyte, the total number of patients is 122 in each omic level.

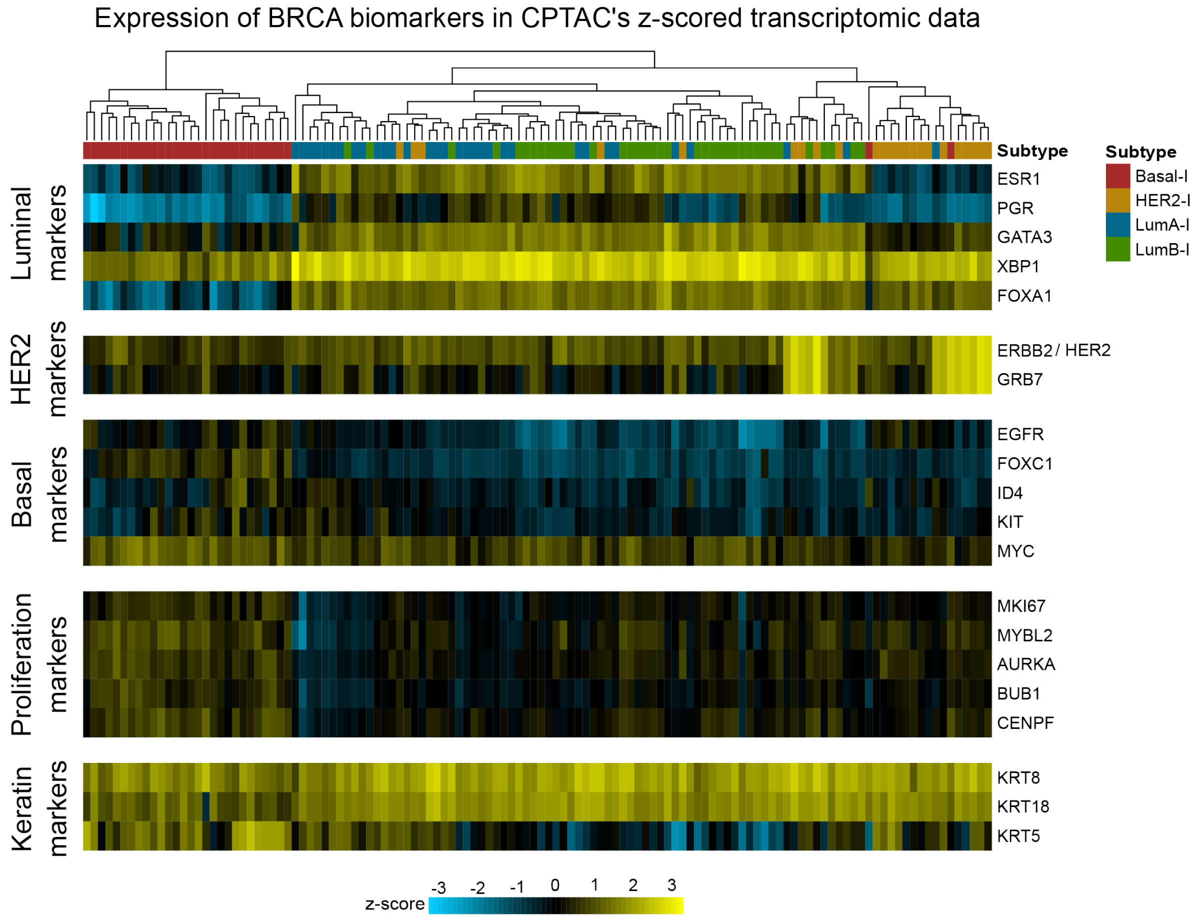

Appendix Figure S4 Nolan Biomarkers in CPTAC data. Heatmap displaying the z-score values of a selection of breast cancer biomarkers in CPTAC patients. The heatmap shows distinct expression patterns across different subtypes (LumA-l, LumB-l, HER2-l, Basal-l) consistent with those observed in the TCGA dataset (Nolan *et al*, 2023)(Hoadley et al, 2014) .

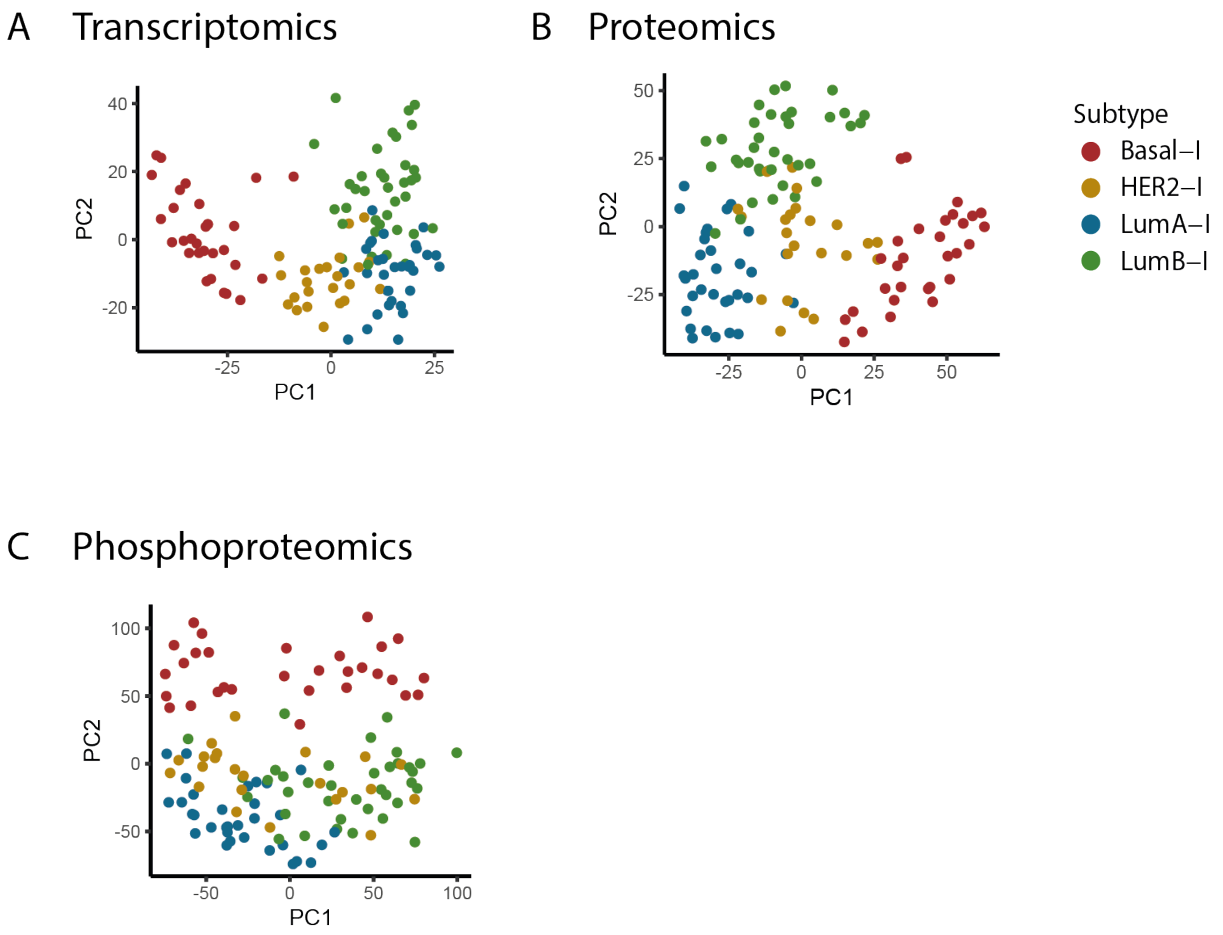

Appendix Figure S5. Subtype stratification at multi-omic levels. Principal component Analysis Protein (PCA) at **A)** Transcriptomic; **B)** Proteomic; and **C)** Phosphoproteomic levels, after data manipulation (STEP1). Subtype color code: Basal-l in red, LumA-l in blue, LumB-l in green, and HER2-l in yellow.

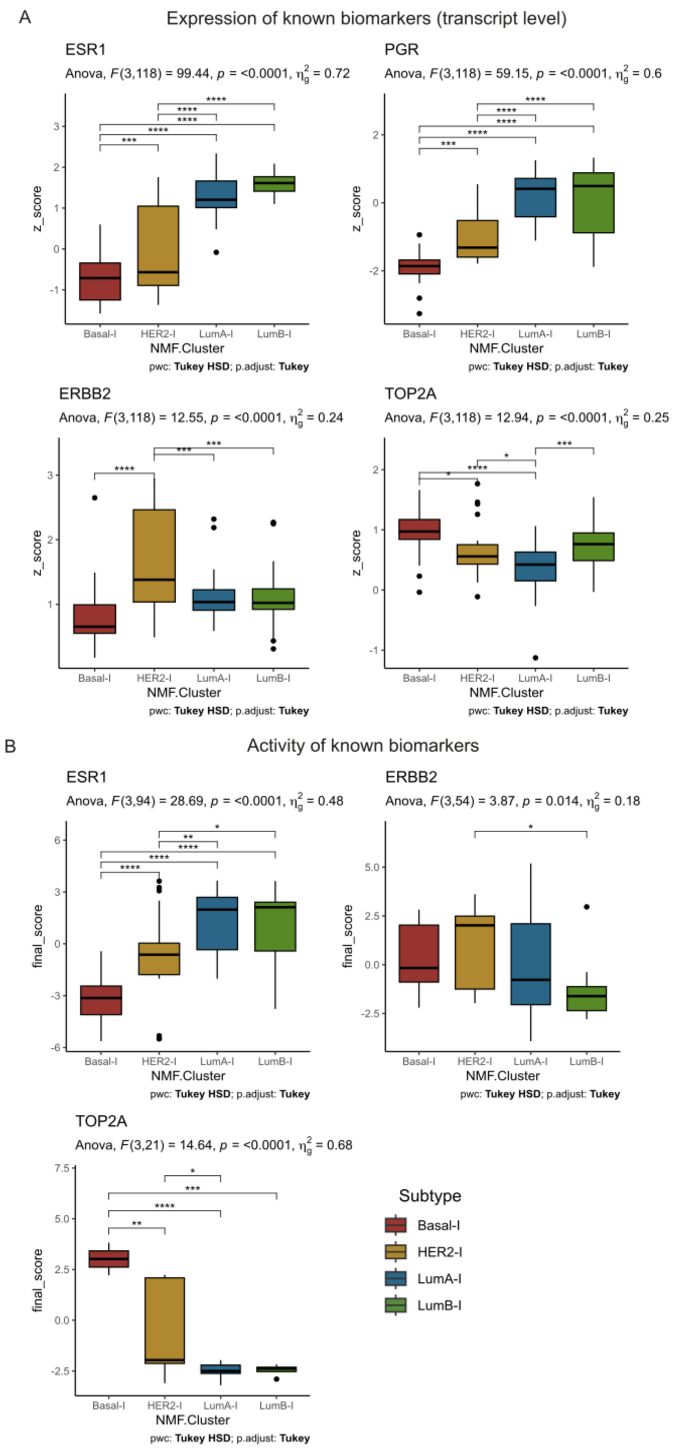

Appendix Figure S6 Subtypes known biomarkers. A) Transcript expression level of known biomarkers in different subtypes, after data manipulation (STEP1). **B)** Protein activity level of known biomarkers in different subtypes, as inferred in STEP2 (ESR1: estrogen receptor, PGR: progesterone receptor, ERBB2/HER2: human epidermal growth factor receptor 2, TOP2A: proliferative biomarker). Subtype color code: Basal-l in red, LumA-l in blue, LumB-l in green, and HER2-l in yellow. Significance was calculated by applying ANOVA test, Benjamini-Hochberg adjusted p-value: *<0.05, **<0.01, ***<0.001, ****<0.0001.

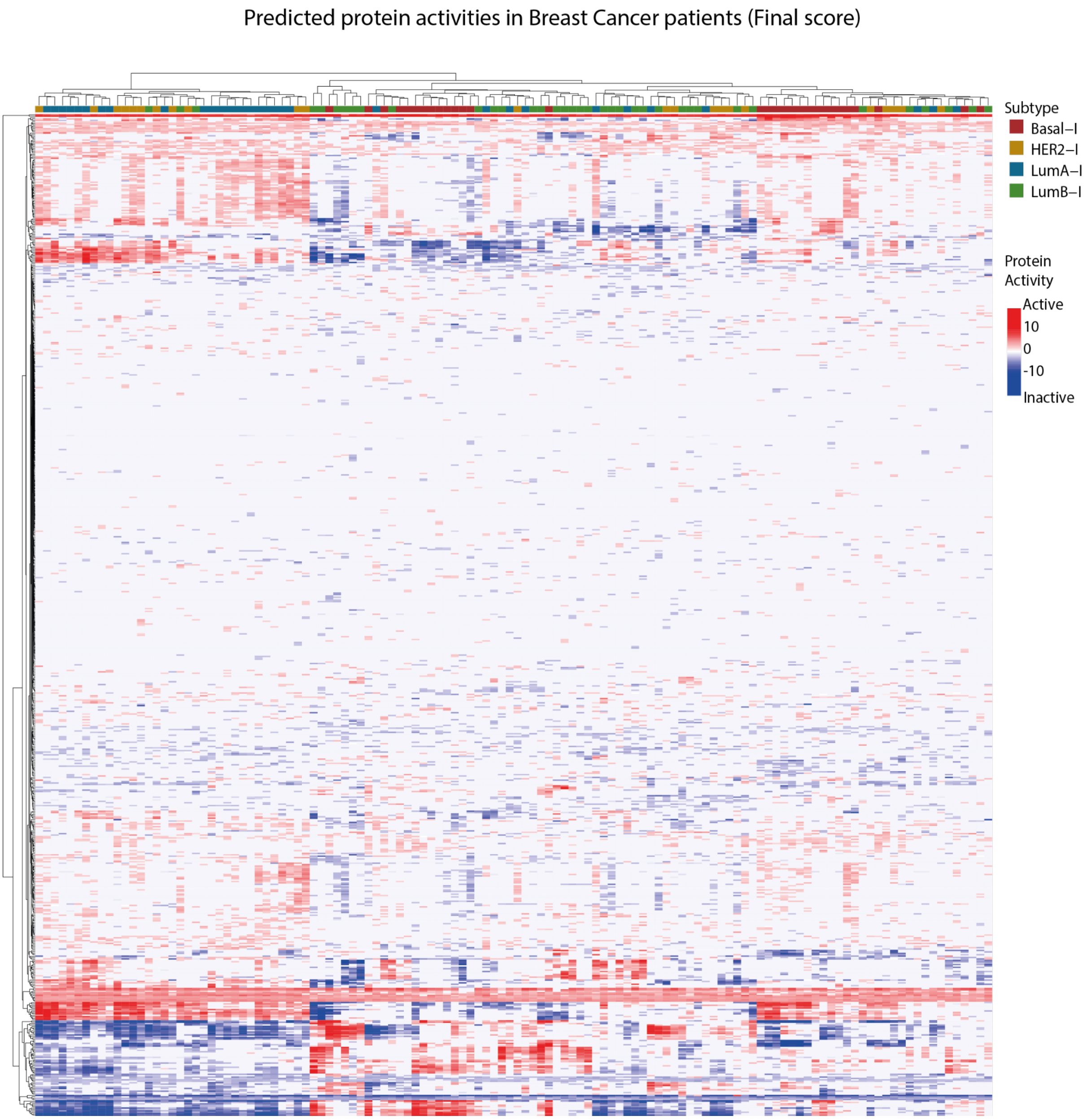

Appendix Figure S7 Predicted protein activities in the Breast Cancer cohort (Final score). Heatmap showing the activation level of individual proteins in individual patients, as derived from the final score, which estimates activity from the multi-omic profiles (STEP2 of the *PatientProfiler* pipeline). Red and blue indicate active and inactive proteins, respectively. Patients and genes are clustered (as shown in the dendrograms) accordingly, using Euclidean distance. Patients are annotated with subtype classification: Basal-l in red, LumA-l in blue, LumB-l in green, and HER2-l in yellow.

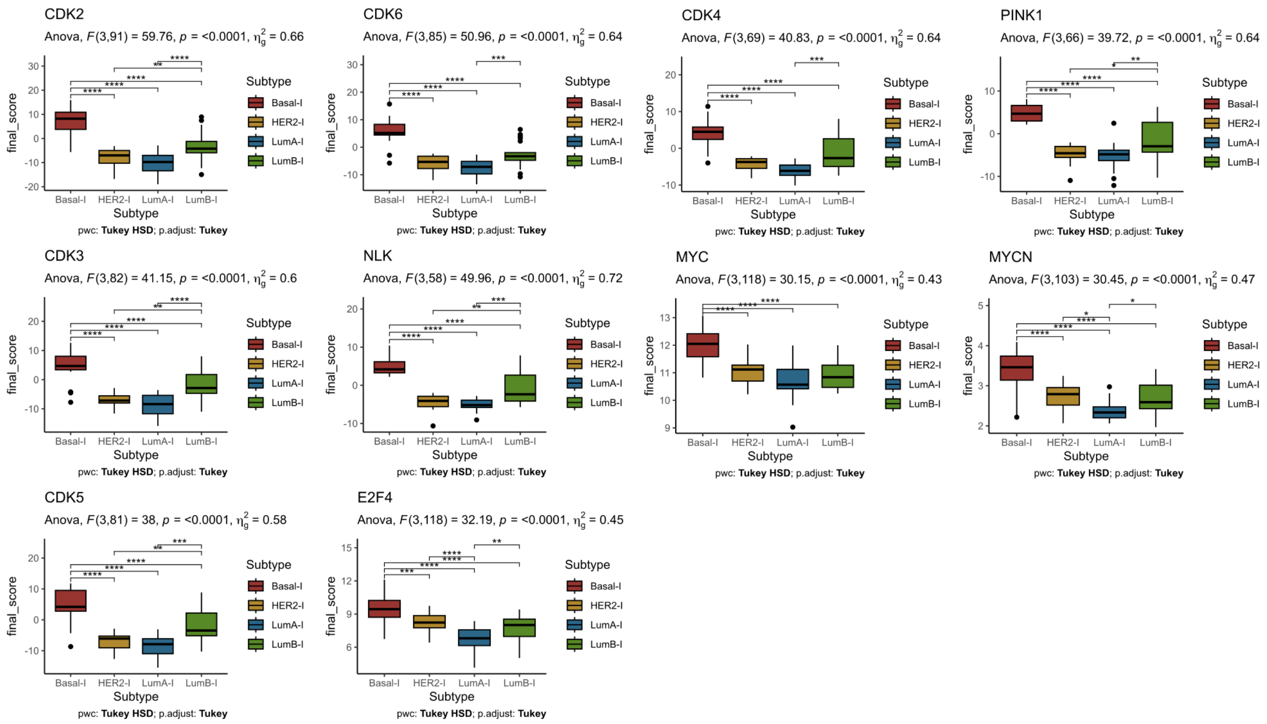

Appendix Figure S8 Top deregulated proteins among subtypes. Protein activity level of top ten deregulated proteins among subtypes. Protein activity (final_score) was inferred in STEP2. Subtype color code: Basal-l in red, LumA-l in blue, LumB-l in green, and HER2-l in yellow. Significance was calculated by applying ANOVA test, Benjamini-Hochberg adjusted p-value: *<0.05, **<0.01, ***<0.001, ****<0.0001.

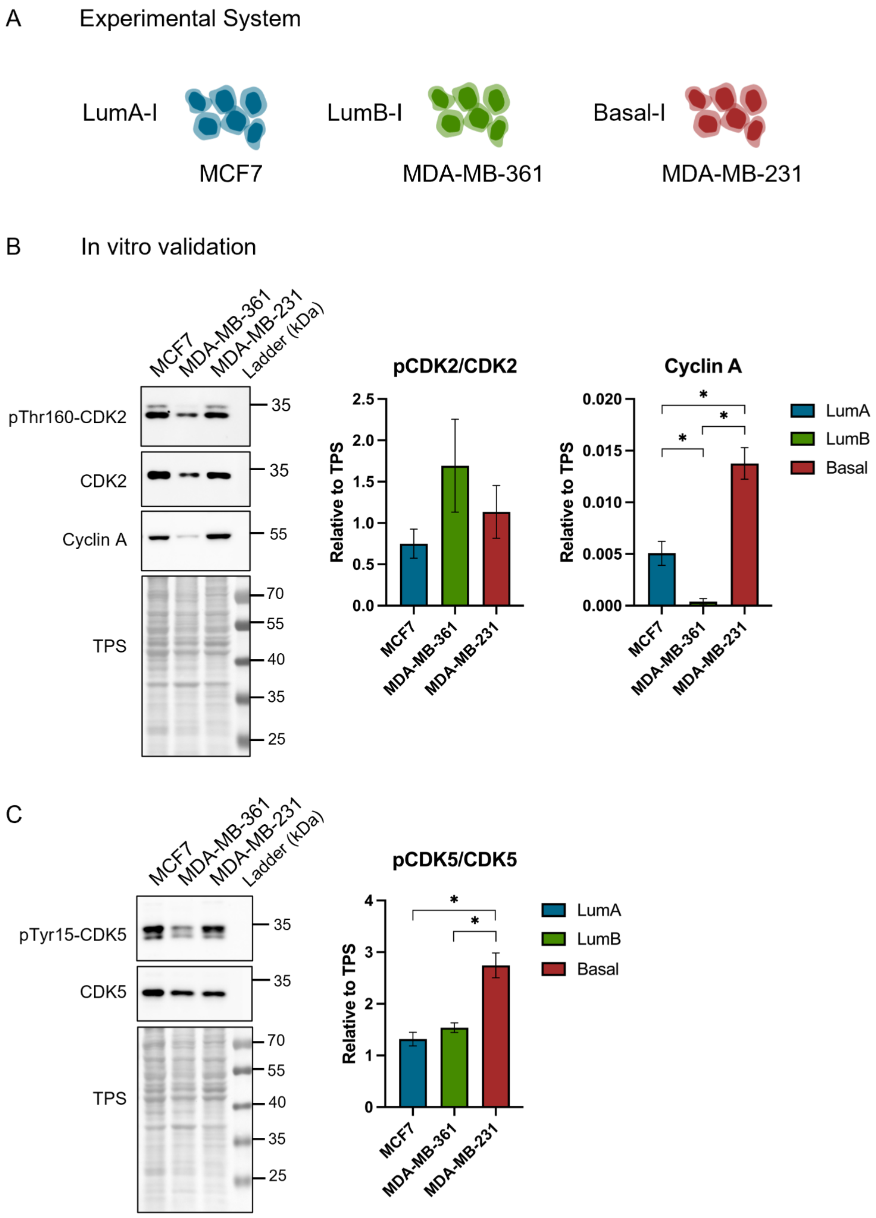

Appendix Figure S9 In vitro validation, STEP 1-2. A) Cartoons representing the experimental systems: Basal-l, LumA-l and LumB-l subtypes were represented by MDA-MB-231, MCF7 and MDA-MB-361 cell lines, respectively. **B)** Experimental validation of CDK2 activity. Western blots showing protein levels of CDK2, phosphorylation levels of activatory p-Thr160 CDK2, and protein levels of CyclinA in MDA-MB-231 (red bars), MDA-MB-361 (green bars) and MCF7 (blue bars). The histogram plots represent the western blot quantification of three independent biological replicates, relative to the Total Protein Staining (TPS). **C)** Experimental validation of CDK5 activity. Western blots showing protein levels of CDK5, and phosphorylation levels of activatory p-Tyr15 CDK5, in MDA-MB-231 (red bars), MDA-MB-361 (green bars) and MCF7 (blue bars). The histogram graph represents the relative to TPS western blot quantification of three independent biological replicates. Significance was calculated by applying Student's T-test, p-value: *<0.05, **<0.01, ***<0.001, ****<0.0001. Full uncropped gels relative to the blots shown in this figure have been provided in **Appendix Figure S21-S22**.

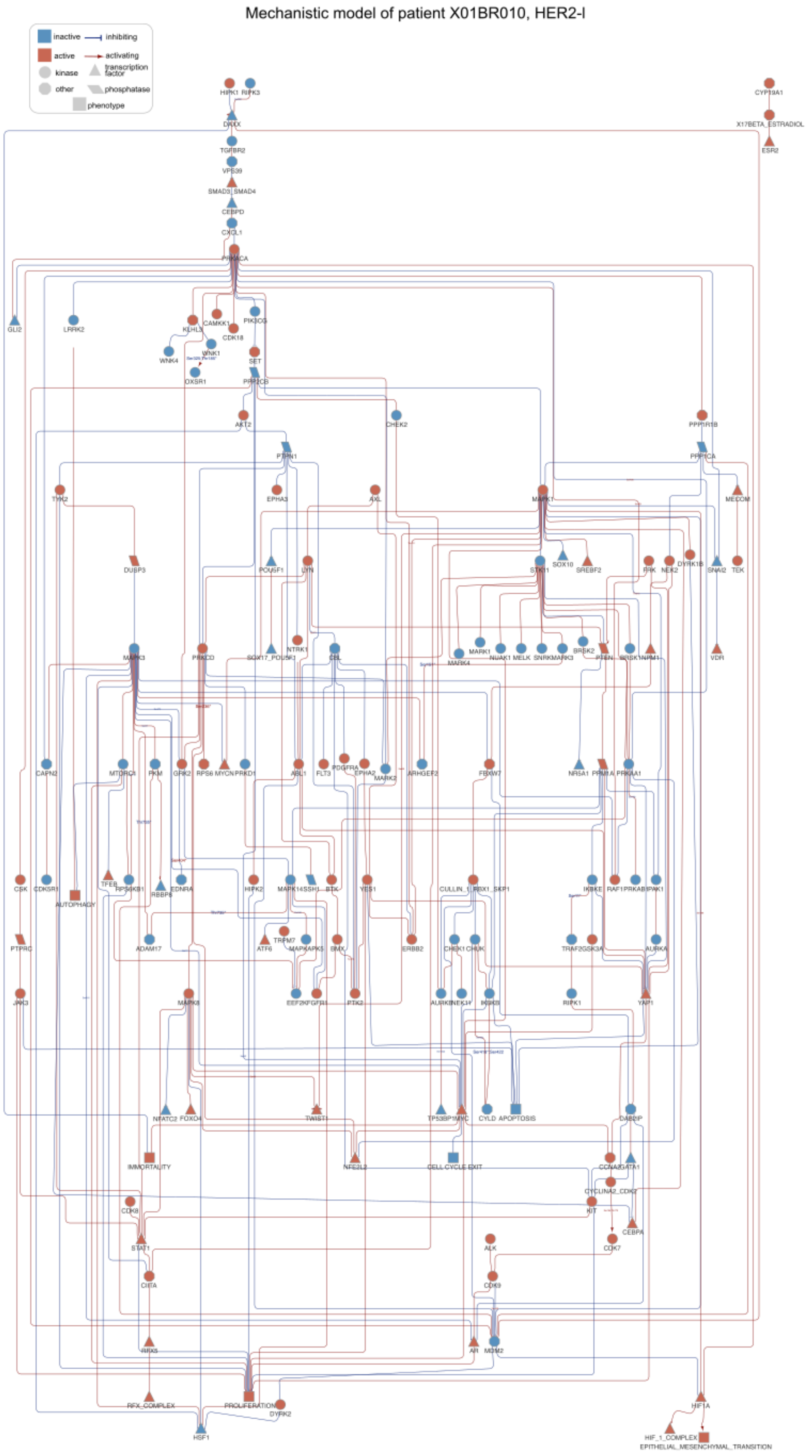

Appendix Figure S10 Example of a patient-specific mechanistic model. Mechanistic model of the HER2-l patient X01BR010. Red and blue nodes represent active and inactive proteins, respectively; red arrows and t-shaped edges represent activatory and inhibitory relationships, respectively. Detected phosphorylated residues are shown as edge labels (e.g., Ser668). NDEx LINK: https://www.ndexbio.org/#/networkset/1387170f-4c20-11f0-a218-005056ae3c32?accesskey=2dfafd3f0a02204ef789f7b4b93741fa193013ae7de552eb60e1ea90e187302a

A

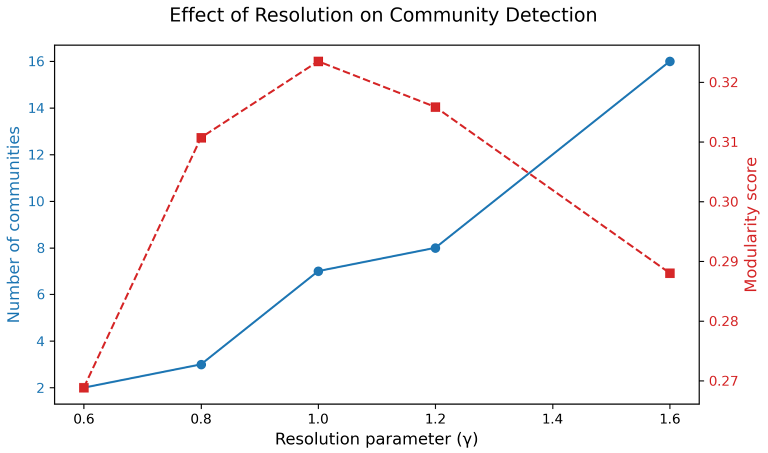

B

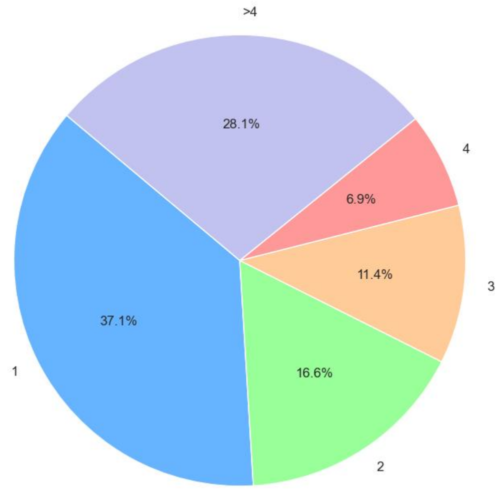

Appendix Figure S11 Tuning of Resolution Parameter. A) Effect of resolution parameter (γ) on community detection. To empirically tune the resolution parameter, we performed community detection across a range of resolution values and evaluated the resulting partitions based on their modularity scores. The number of detected communities (left y-axis, blue line) increases with γ, while the modularity score (right y-axis, red dashed line) peaks around 1.0. The resolution value corresponding to the highest modularity score was selected for downstream analysis (γ=0.99). **B**) Distribution of interaction-nodes. The pie chart shows the percentage of interaction-nodes based on how many patients share them. Each slice corresponds to the proportion of interactions appearing in exactly 1, 2, 3, or 4 patients, as well as those shared by more than 4 patients (>4).

Breast Cancer detected communities, summary

Community	# Nodes	#Edges	#Patients	Ambiguous Nodes (%)	Derived Signature	# Genes in Signature
Community 1 (CL1)	141	171	15	3.5	Signature 1	12
Community 2 (CL2)	183	234	27	10.3	Signature 2	11
Community 3 (CL3)	76	66	7	1.3	Signature 3	28
Community 4 (CL4)	120	131	15	1.6	Signature 4	16
Community 5 (CL5)	176	233	25	6.8	Signature 5	27
Community 6 (CL6)	166	156	17	4.2	Signature 6	11
Community 7 (CL7)	123	110	16	3.2	Signature 7	21

Appendix Figure S12 Breast cancer communities, summary data. Information associated to each community: number of nodes, number of edges, number of patients, percentage of ambiguous nodes (i.e. nodes that appear as both active and inactive in the relative community), associated transcriptomic signatures, number of genes in the transcriptomic signatures.

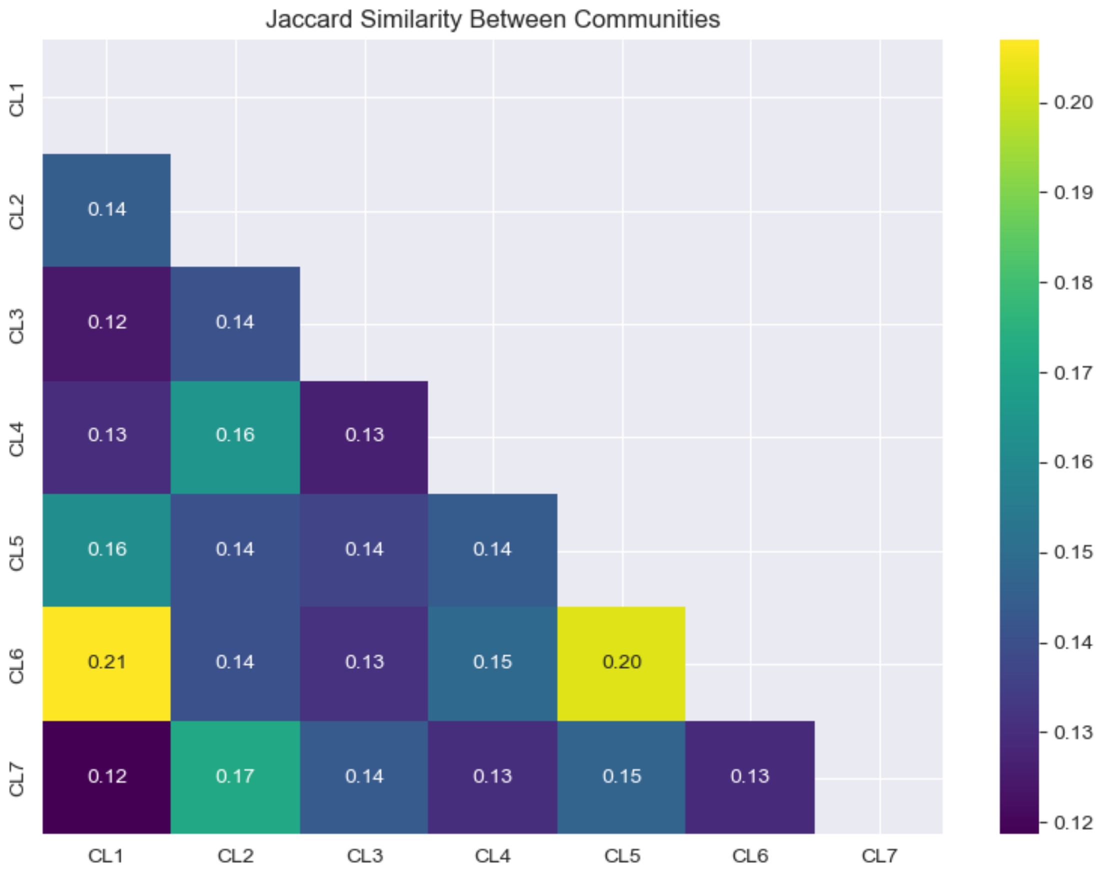

Appendix Figure S13 Similarity across Communities. The heatmap shows the overlap between detected communities (Jaccard's similarity). Each value represents the Jaccard index between pairs of communities, calculated from the set of genes assigned to each. The generally low similarity scores suggest a significative structural difference across communities.

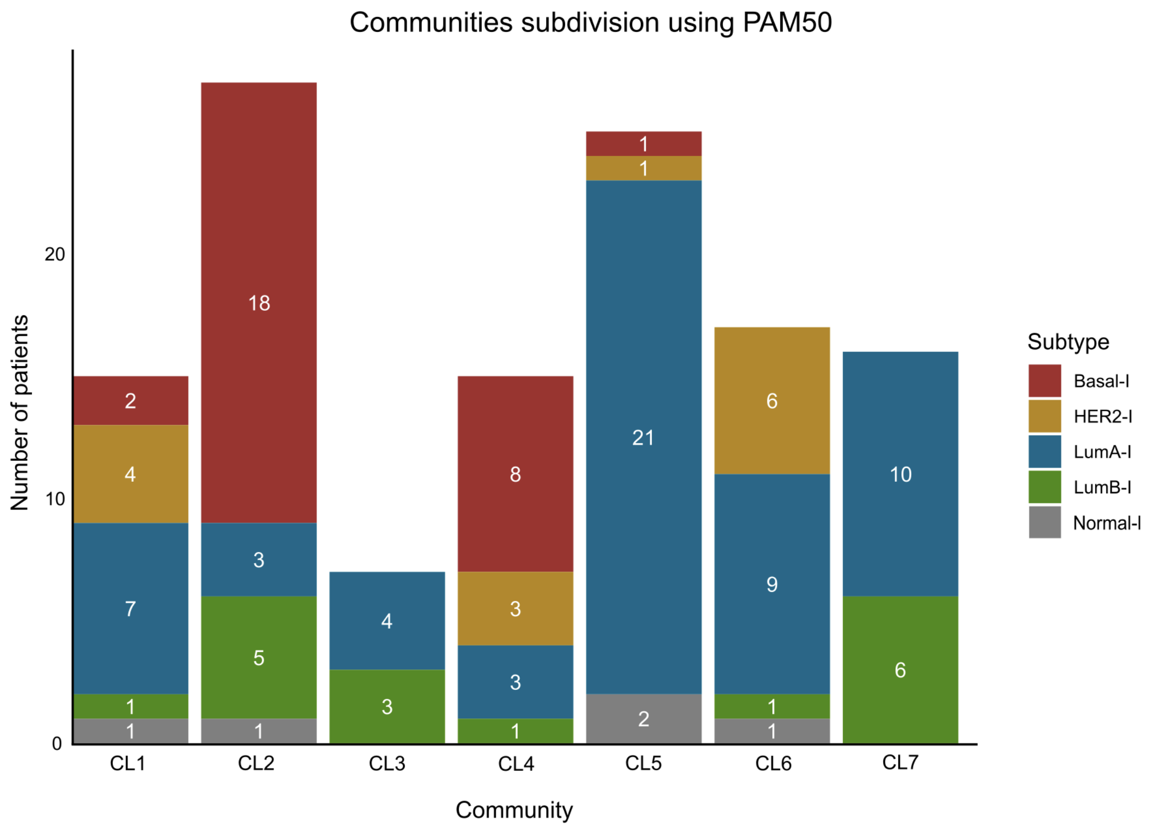

Appendix Figure S14 Communities' subtype composition (PAM50). The cohort of 122 Breast Cancer patients is divided into seven communities (CL1-7). Bar plots depicting the subtype composition of each community. Subtype color code refers to the PAM50 classification (Kensler *et al*, 2019), as derived from the original publication (Krug *et al*, 2020): Basal-l in red, LumA-l in blue, LumB-l in green, HER2-l in yellow, and Normal-l in gray.

Expression level of community-derived transcriptomic signatures

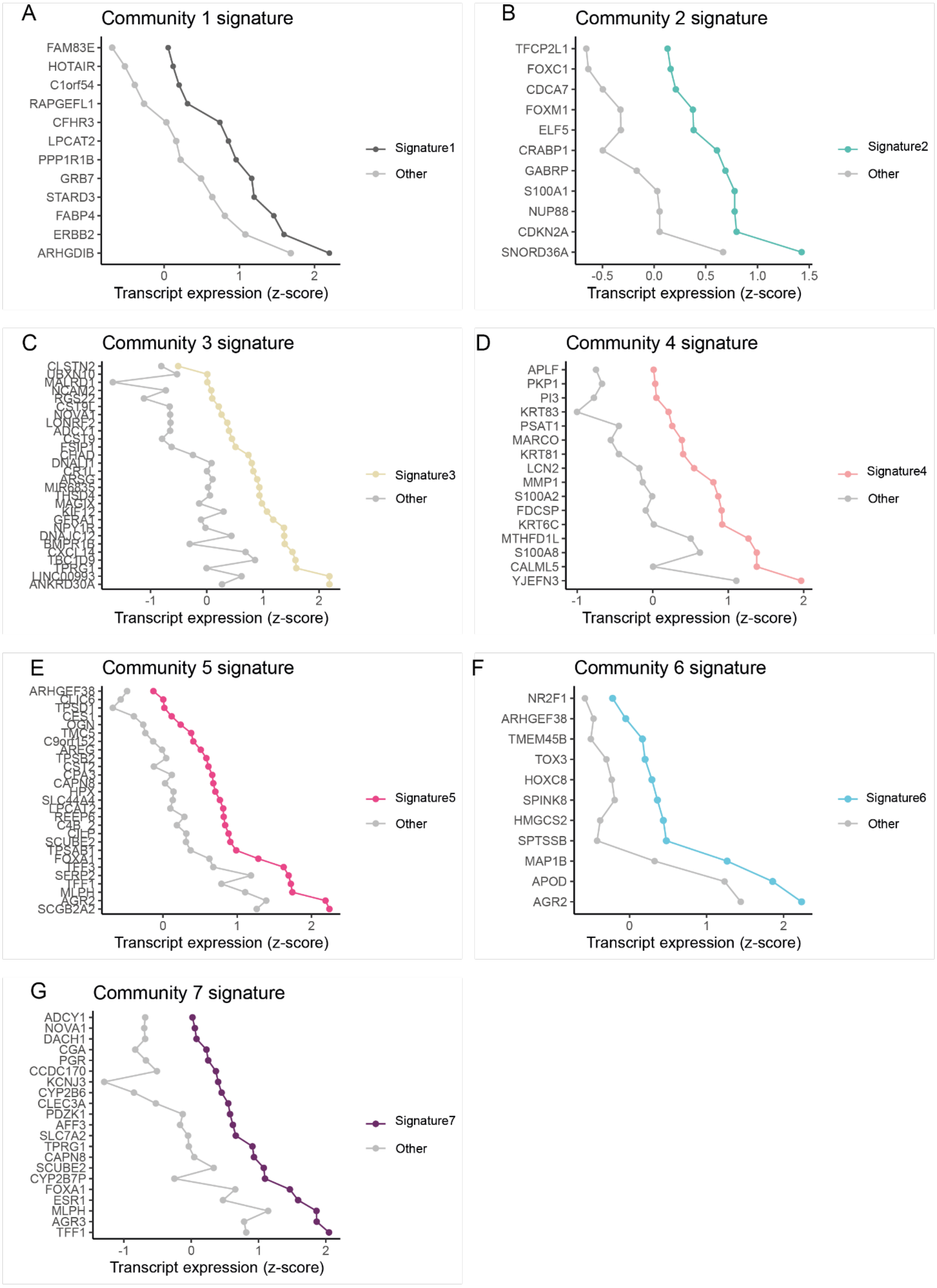

Appendix Figure S15 Expression level of the signatures. A-G) Plots showing the average expression level (z-score, as derived in STEP1) of each gene in each transcriptomic signature (as derived in STEP4), relative to the patients belonging to the community (CL1-7) with respect to the rest of the cohort (Other - in gray). Plots refer to (**A**) Community 1; (**B**) Community 2; (**C**) Community 3; (**D**) Community 4; (**E**) Community 5; (**F**) Community 6; and (**G**) Community 7.

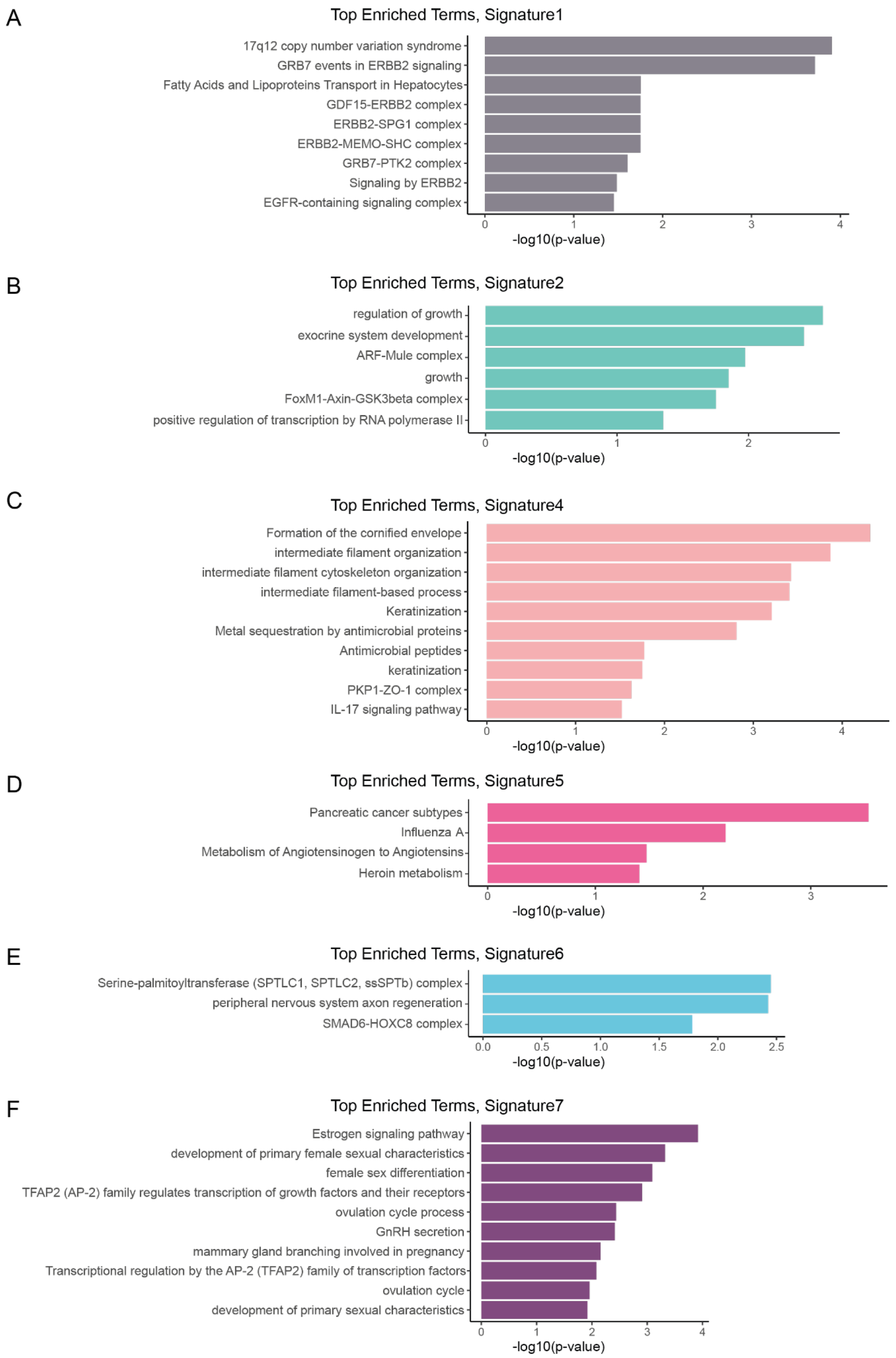

Appendix Figure S16 Terms overrepresentation analysis of community-derived transcriptomic signatures. A-F) Pathway and biological process terms over-represented in each community-derived transcriptomic signature. The plots list the GO: Biological Processes, Reactome, WikiPathways, and KEGG Pathways (Gene Ontology Consortium *et al*, 2023; Kanehisa *et al*, 2017; Slenter *et al*, 2018; Gillespie *et al*, 2022) significantly enriched in the signature. Only the top 10 significantly (Bonferroni-adjusted *P*-value < 0.05) over-represented terms are shown. Analyses were carried out by using the gProfiler2 software (Kolberg *et al*, 2020), using the entire Human Proteome as a background. Plots are relative to (**A**) Signature 1; (**B**) Signature 2; (**C**) Signature 4; (**D**) Signature 5; (**E**) Signature 6; (**F**) Signature 7.

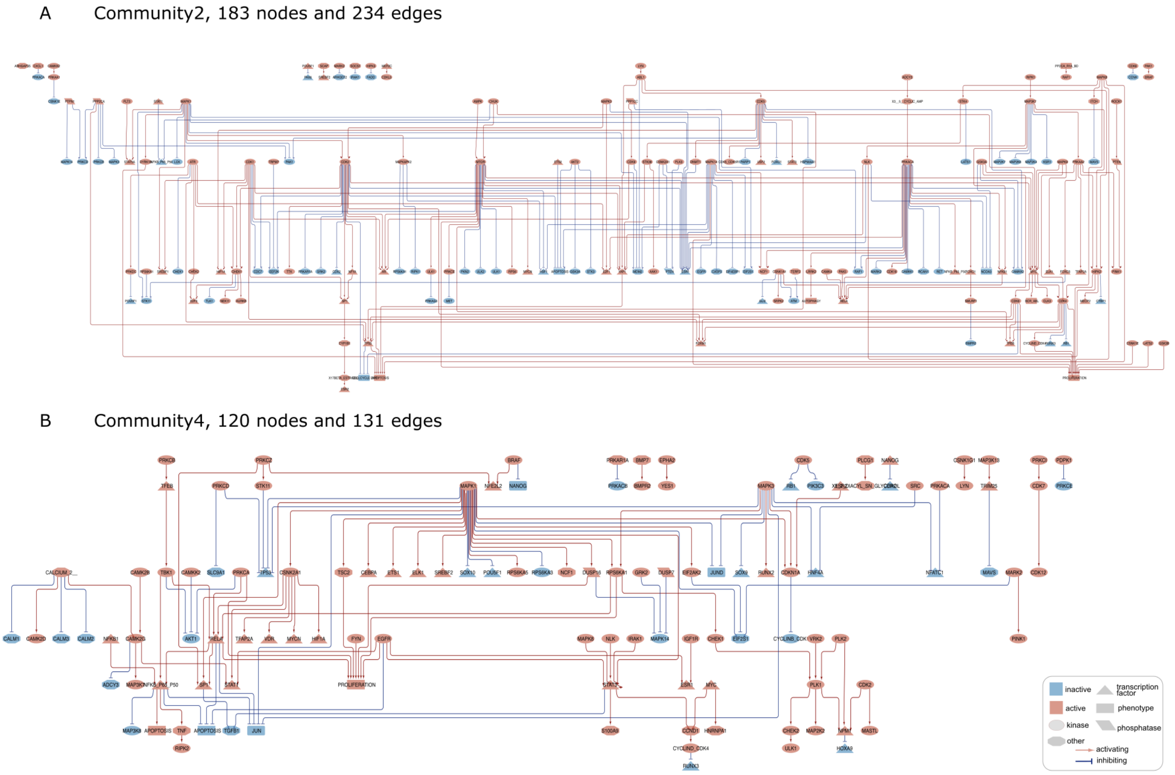

Appendix Figure S17 Basal-l communities. Mechanistic models generated with *PatientProfiler*. The graph depicts the functional graph associated with **A)** Community 2; and **B)** Community 4. Red and blue nodes represent active and inactive proteins, respectively; red arrows and t-shaped edges represent activatory and inhibitory relationships, respectively. NDEx LINK:
https://www.ndexbio.org/#/networkset/dd734d60-4c24-11f0-a218-005056ae3c32?accesskey=573cb8bc7f0977857ac19a6a6b65c231320fe8baced6a756aaf32570e0a8fa18

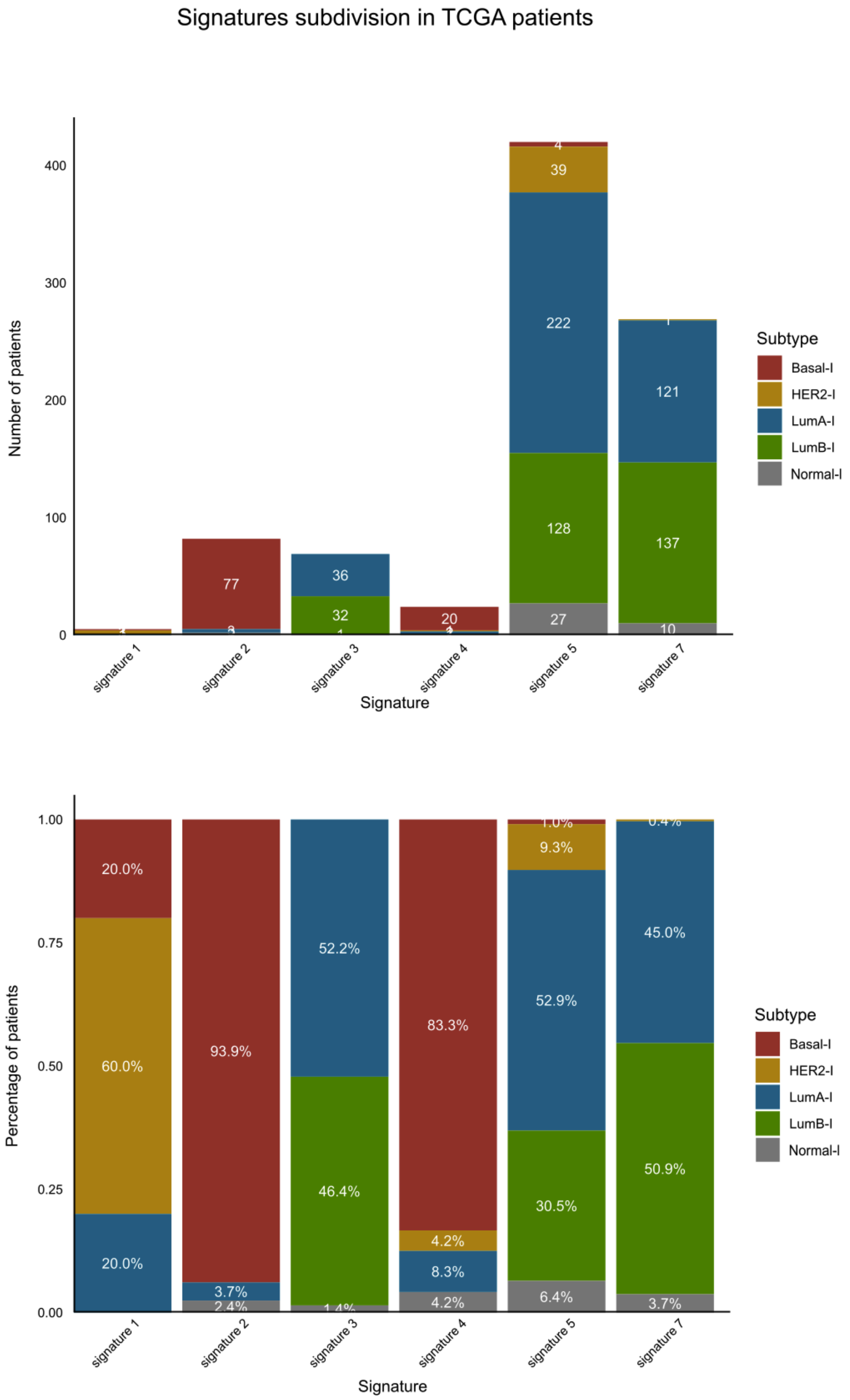

Appendix Figure S18 Signature-based stratification of TCGA Breast Cancer patients. Breast cancer patients-derived transcriptomic data from The Cancer Genome Atlas (TCGA) (Koboldt *et al*, 2012) was used in combination (gene set enrichment analysis - GSEA) with community-derived transcriptomic signatures to stratify TCGA patients in seven communities (Signature 1-7) (Benjamini-Hochberg correction, adjusted p-value < 0.01, NES >0). No enriched patients for signature 6. Breast cancer subtypes for TCGA patients were retrieved from the PAM50 classification (Kensler *et al*, 2019) reported by (Lehmann *et al*, 2021). **A)** Bar plots depicting the subtype composition in number of patients for each signature. **B)** Bar plots showing the percentage distribution of subtypes within each signature. Subtype color code: Basal-l in red, LumA-l in blue, LumB-l in green, HER2-l in yellow, Normal-l in gray.

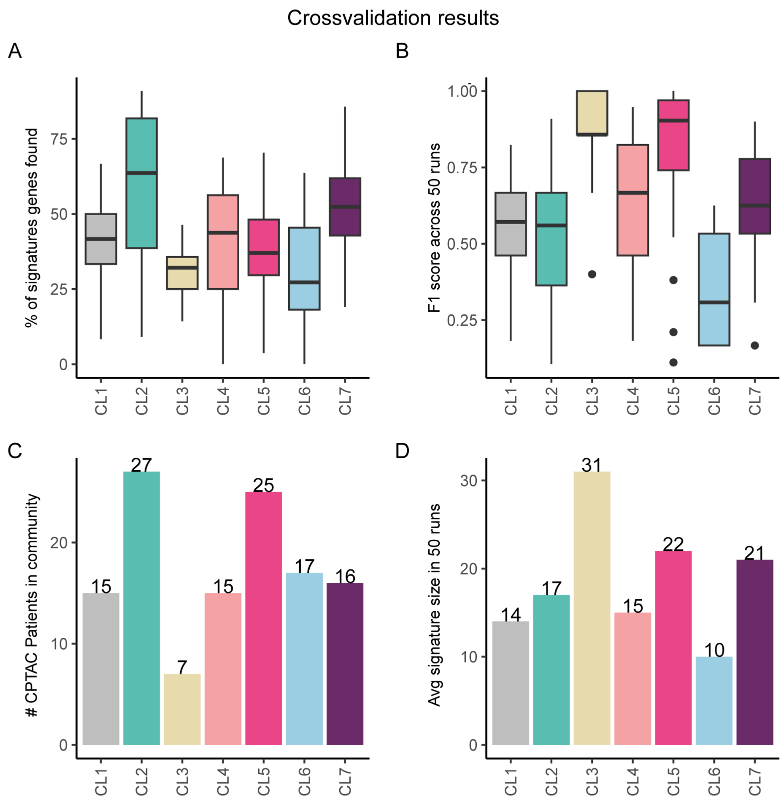

Appendix Figure S19 Cross-validation results for community-specific classification performance. A) Percentage of signature genes recovered in each of 50 cross-validation runs per community. **B**) F1 score distributions showing classification performance across runs. **C**) Number of CPTAC patients assigned to each community in the full run. **D)** Average number of genes included in the signature for each community across the 50 cross-validation runs.

	Method	Input data type	Reference	Stratification	Modelling		Type of graph		Protein-activity estimation	Sample-specific network/model
					Mechanistic	Dynamic	Directed	Undirected		
data-driven methods	Kmeans	Multi-omics	Ikotun et al, 2023							
		PCA	Hotelling, 1933	X						
		tSNE	Maaten & Hinton, 2008	X						
		MOFA	Argelaguet et al, 2018	X						
	Hierarchical Clustering	Multi-omics	Murtagh & Contreras, 2017	X						
		PCA	Hotelling, 1933	X						
		tSNE	Maaten & Hinton, 2008	X						
		MOFA	Argelaguet et al, 2018	X						
graph-based methoc	*PatientProfiler*	Multi-omics	This study	X	X		X		X	X
	iSNP	SNPs	Brooks-Warburton et al, 2022	X			X			X
	netDx*	Multi-omics and clinical	Pai et al, 2019	X			X			
	COSMOS	Multi-omics	Dugourd et al, 2021		X		X		X	X
	PROFILE	Transcriptomic	Montagud et al, 2022			X	X		X	X
	pCHIP	Multi-omics	Drake et al, 2016					X	X	X

*machine leanring

Appendix Figure S20 Overview of computational tools compared with *PatientProfiler*. The table details the input type (whole omics or the output of different dimensionality reduction techniques), article reference (numbered according to Supplementary Material), patients' stratification ability, graph-based output (directed/undirected), code availability, sample-specific output, inclusion in quantitative comparison, and category classification based on these features: Category I are tools that that generate sample-specific, mechanistic models; Category II are tools that leverage multi-omic data to stratify patients and derive prognostic transcriptomic signatures.

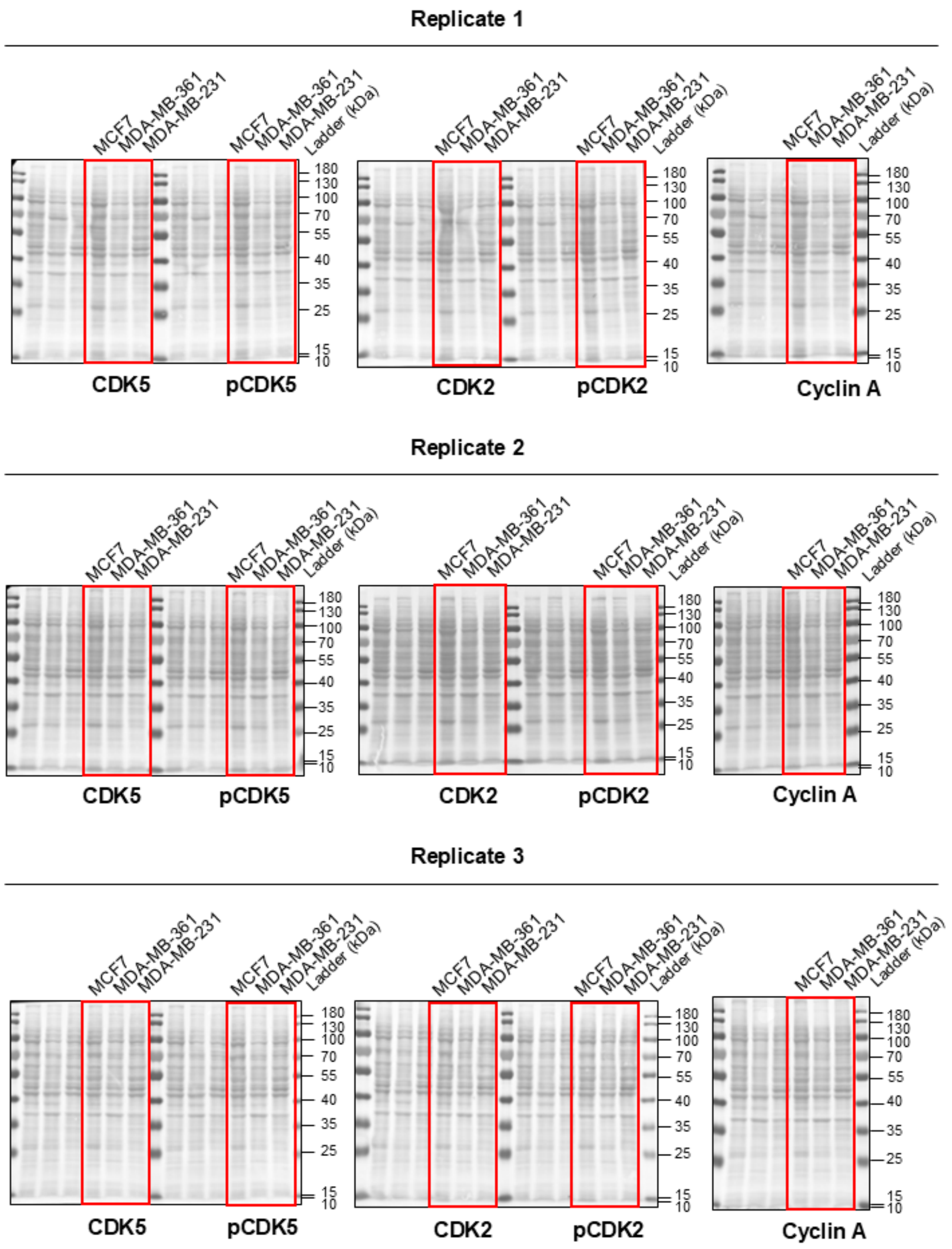

Appendix Figure S21 Total Protein Staining. Uncropped gels showing total protein staining (TPS) with ready-to-use Ponceau S solution for the three biological replicates used in target normalization are presented (refer to **Supplementary Figure 9**). Red rectangles indicate the lanes corresponding to the samples of the three biological replicates for each respective target, as indicated in the figure. The full-lane Ponceau S signal was quantified and used for normalization of the target signals. Quantification was performed using *Image Lab* software version 6.1 (Bio-Rad).

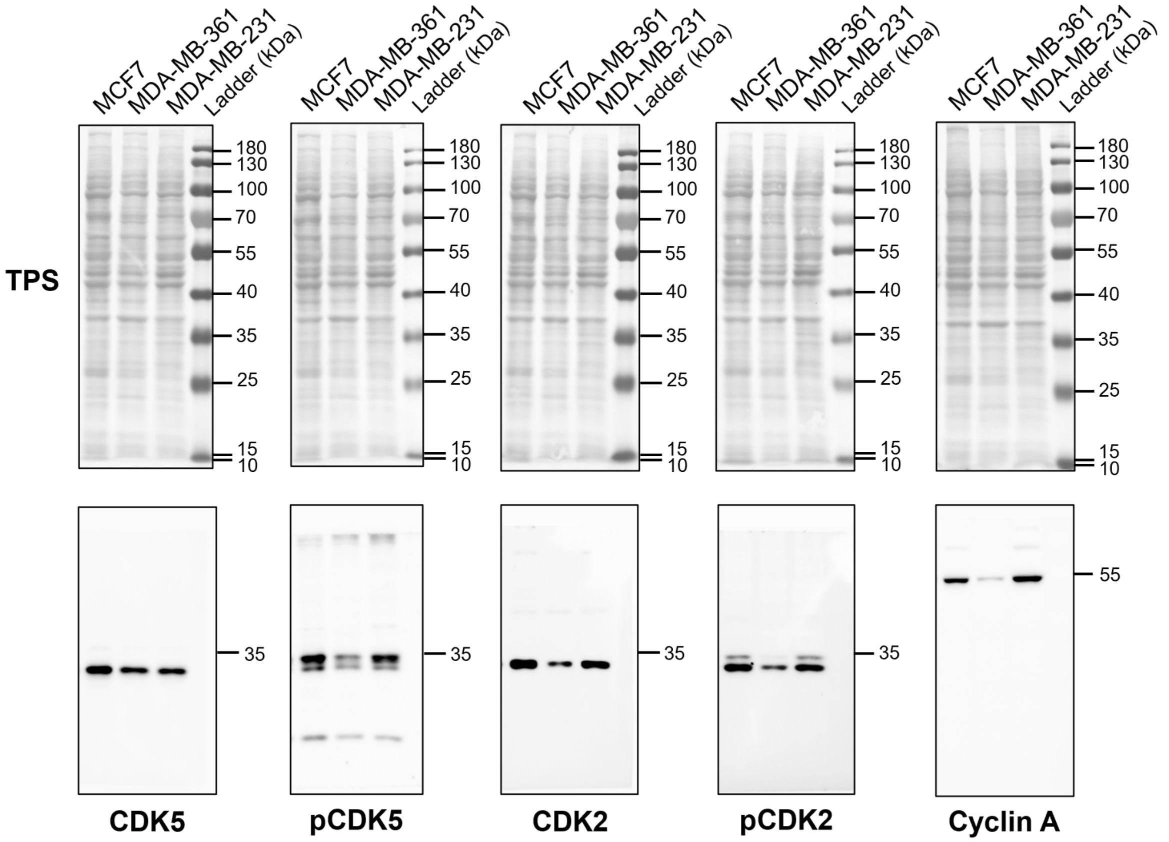

Appendix Figure S22. Uncropped versions of the blots shown in **Appendix Figure S9** are provided here. These images correspond to one of the three biological replicates used for quantification. Full gel images are shown for each target protein and loading control, with molecular weight ladders indicated, confirming the absence of significant off-target bands detected outside the specific molecular weight range corresponding to proteins of interest.

Bibliography

Alvarez MJ, Shen Y, Giorgi FM, Lachmann A, Ding BB, Ye BH & Califano A (2016) Functional characterization of somatic mutations in cancer using network-based inference of protein activity. *Nat Genet* 48: 838–847

Argelaguet R, Velten B, Arnol D, Dietrich S, Zenz T, Marioni JC, Buettner F, Huber W & Stegle O (2018) Multi-Omics Factor Analysis-a framework for unsupervised integration of multi-omics data sets. *Mol Syst Biol* 14: e8124

Brooks-Warburton J, Modos D, Sudhakar P, Madgwick M, Thomas JP, Bohar B, Fazekas D, Zoufir A, Kapuy O, Szalay-Beko M, *et al* (2022) A systems genomics approach to uncover patient-specific pathogenic pathways and proteins in ulcerative colitis. *Nat Commun* 13: 2299

Chakravarty D, Gao J, Phillips SM, Kundra R, Zhang H, Wang J, Rudolph JE, Yaeger R, Soumerai T, Nissan MH, *et al* (2017) OncoKB: A Precision Oncology Knowledge Base. *JCO Precis Oncol* 2017: PO.17.00011

Drake JM, Paull EO, Graham NA, Lee JK, Smith BA, Titz B, Stoyanova T, Faltermeier CM, Uzunangelov V, Carlin DE, *et al* (2016) Phosphoproteome Integration Reveals Patient-Specific Networks in Prostate Cancer. *Cell* 166: 1041–1054

Dugourd A, Kuppe C, Sciacovelli M, Gjerga E, Gabor A, Emdal KB, Vieira V, Bekker-Jensen DB, Kranz J, Bindels EMJ, *et al* (2021) Causal integration of multi-omics data with prior knowledge to generate mechanistic hypotheses. *Mol Syst Biol* 17: e9730

Gene Ontology Consortium, Aleksander SA, Balhoff J, Carbon S, Cherry JM, Drabkin HJ, Ebert D, Feuermann M, Gaudet P, Harris NL, *et al* (2023) The Gene Ontology knowledgebase in 2023. *Genetics* 224: iyad031

Gillespie M, Jassal B, Stephan R, Milacic M, Rothfels K, Senff-Ribeiro A, Griss J, Sevilla C, Matthews L, Gong C, *et al* (2022) The reactome pathway knowledgebase 2022. *Nucleic Acids Res* 50: D687–D692

Hoadley KA, Andre F, Ellis MJ, Perou CM (2014) Breast cancer intrinsic subtypes. *Poster Nat. Rev. Clin. Oncol.* https://www.nature.com/documents/nrclinonc_posters_breastcancer.pdf

Hornbeck PV, Zhang B, Murray B, Kornhauser JM, Latham V & Skrzypek E (2015) PhosphoSitePlus, 2014: mutations, PTMs and recalibrations. *Nucleic Acids Res* 43: D512-520

Hotelling H (1933) Analysis of a complex of statistical variables into principal components. *J Educ Psychol* 24: 417–441

Iannuccelli M, Vitriolo A, Licata L, Lo Surdo P, Contino S, Cheroni C, Capocefalo D, Castagnoli L, Testa G, Cesareni G, *et al* (2023) Curation of causal interactions mediated by genes associated with autism accelerates the understanding of gene-phenotype relationships underlying neurodevelopmental disorders. *Mol Psychiatry*

Ikotun AM, Ezugwu AE, Abualigah L, Abuhaija B & Heming J (2023) K-means clustering algorithms: A comprehensive review, variants analysis, and advances in the era of big data. *Inf Sci* 622: 178–210

Kanehisa M, Furumichi M, Tanabe M, Sato Y & Morishima K (2017) KEGG: new perspectives on genomes, pathways, diseases and drugs. *Nucleic Acids Res* 45: D353–D361

Kensler KH, Sankar VN, Wang J, Zhang X, Rubadue CA, Baker GM, Parker JS, Hoadley KA, Stancu AL, Pyle ME, *et al* (2019) PAM50 Molecular Intrinsic Subtypes in the Nurses' Health Study Cohorts. *Cancer Epidemiol Biomark Prev Publ Am Assoc Cancer Res Cosponsored Am Soc Prev Oncol* 28: 798–806

Koboldt DC, Fulton RS, McLellan MD, Schmidt H, Kalicki-Veizer J, McMichael JF, Fulton LL, Dooling DJ, Ding L, Mardis ER, *et al* (2012) Comprehensive molecular portraits of human breast tumours. *Nature* 490: 61–70

Kolberg L, Raudvere U, Kuzmin I, Vilo J & Peterson H (2020) gprofiler2 -- an R package for gene list functional enrichment analysis and namespace conversion toolset g:Profiler. *F1000Research* 9: ELIXIR-709

Krug K, Jaehnig EJ, Satpathy S, Blumenberg L, Karpova A, Anurag M, Miles G, Mertins P, Geffen Y, Tang LC, *et al* (2020) Proteogenomic Landscape of Breast Cancer Tumorigenesis and Targeted Therapy. *Cell* 183: 1436-1456.e31

Lehmann BD, Colaprico A, Silva TC, Chen J, An H, Ban Y, Huang H, Wang L, James JL, Balko JM, *et al* (2021) Multi-omics analysis identifies therapeutic vulnerabilities in triple-negative breast cancer subtypes. *Nat Commun* 12: 6276

Liu A, Trairatphisan P, Gjerga E, Didangelos A, Barratt J & Saez-Rodriguez J (2019) From expression footprints to causal pathways: contextualizing large signaling networks with CARNIVAL. *Npj Syst Biol Appl* 5: 40

Lo Surdo P, Iannuccelli M, Contino S, Castagnoli L, Licata L, Cesareni G & Perfetto L (2023) SIGNOR 3.0, the SIGnaling network open resource 3.0: 2022 update. *Nucleic Acids Res* 51: D631–D637

Maaten L van der & Hinton G (2008) Visualizing Data using t-SNE. *J Mach Learn Res* 9: 2579–2605

Montagud A, Béal J, Tobalina L, Traynard P, Subramanian V, Szalai B, Alföldi R, Puskás L, Valencia A, Barillot E, *et al* (2022) Patient-specific Boolean models of signalling networks guide personalised treatments. *eLife* 11: e72626

Murtagh F & Contreras P (2017) Algorithms for hierarchical clustering: an overview, II. *WIREs Data Min Knowl Discov* 7: e1219

Nolan E, Lindeman GJ & Visvader JE (2023) Deciphering breast cancer: from biology to the clinic. *Cell* 186: 1708–1728

Pai S, Hui S, Isserlin R, Shah MA, Kaka H & Bader GD (2019) netDx: interpretable patient classification using integrated patient similarity networks. *Mol Syst Biol* 15: e8497

Slenter DN, Kutmon M, Hanspers K, Riutta A, Windsor J, Nunes N, Mélius J, Cirillo E, Coort SL, Digles D, *et al* (2018) WikiPathways: a multifaceted pathway database bridging metabolomics to other omics research. *Nucleic Acids Res* 46: D661–D667

Venafra V, Sacco F & Perfetto L (2024) SignalingProfiler 2.0 a network-based approach to bridge multi-omics data to phenotypic hallmarks. *NPJ Syst Biol Appl* 10: 95
