# Supplementary material for: PatientProfiler: building patient-specific signaling models from proteogenomic data
Source: Mol Syst Biol. 2025 Oct 10;21(12):1845–65. doi: 10.1038/s44320-025-00160-y (PMC12672659; doi:10.1038/s44320-025-00160-y)
Supplement: Supplementary file 6 — Source data Fig. 1 [file 44320_2025_160_MOESM6_ESM.zip › Figure 1/1.pdf]

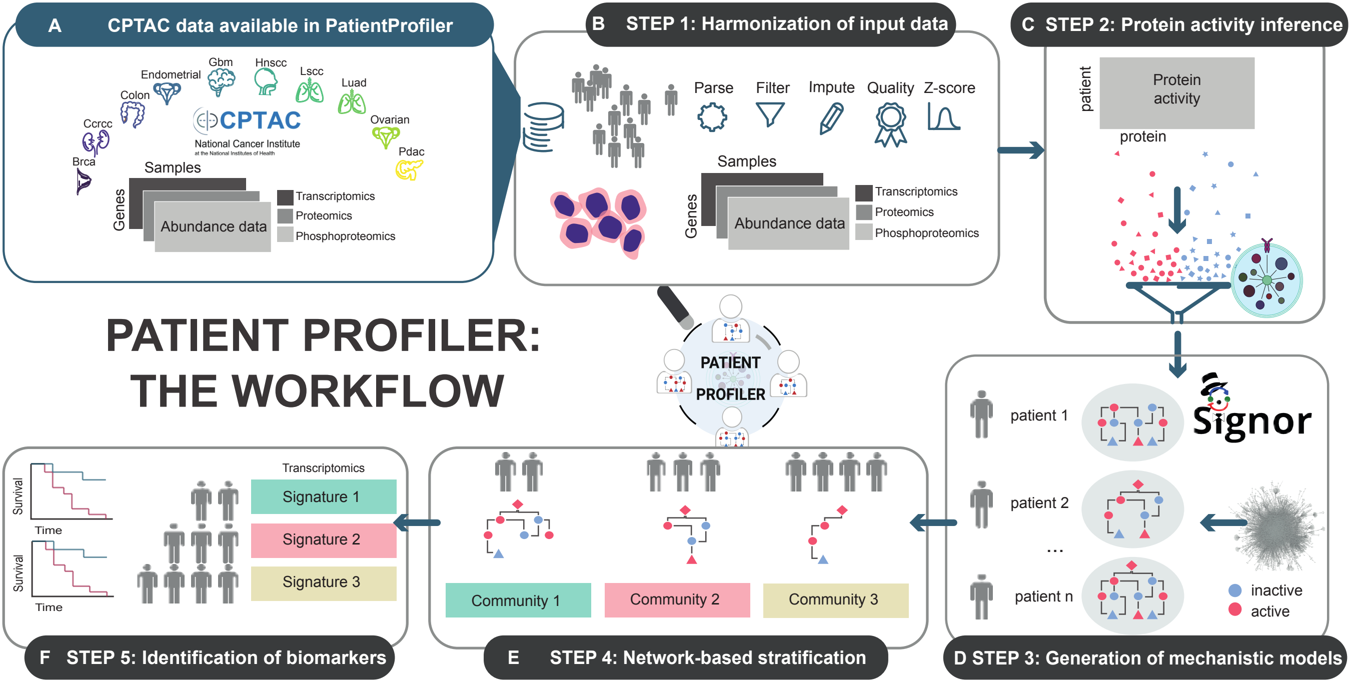

A CPTAC data available in PatientProfiler
Endometrial
Gbm
Hnscc
Lscc
Luad
Colon
Ccrcc
Brca
Ovarian
Pdac
CPTAC
National Cancer Institute
Samples
Genes
Abundance data
Transcriptomics
Proteomics
Phosphoproteomics
B STEP 1: Harmonization of input data
Parse
Filter
Impute
Quality
Z-score
Samples
Genes
Abundance data
Transcriptomics
Proteomics
Phosphoproteomics
C STEP 2: Protein activity inference
patient
Protein activity
protein
PATIENT PROFILER: THE WORKFLOW
PATIENT PROFILER
patient 1
patient 2
...
patient n
Signor
inactive
active
D STEP 3: Generation of mechanistic models
Community 1
Community 2
Community 3
E STEP 4: Network-based stratification
Survival
Time
Survival
Time
Transcriptomics
Signature 1
Signature 2
Signature 3
F STEP 5: Identification of biomarkers
