# Supplementary material for: PatientProfiler: building patient-specific signaling models from proteogenomic data
Source: Mol Syst Biol. 2025 Oct 10;21(12):1845–65. doi: 10.1038/s44320-025-00160-y (PMC12672659; doi:10.1038/s44320-025-00160-y)
Supplement: Supplementary file 7 — Source data Fig. 2 [file 44320_2025_160_MOESM7_ESM.zip › Figure 2/2G/2G.pdf]

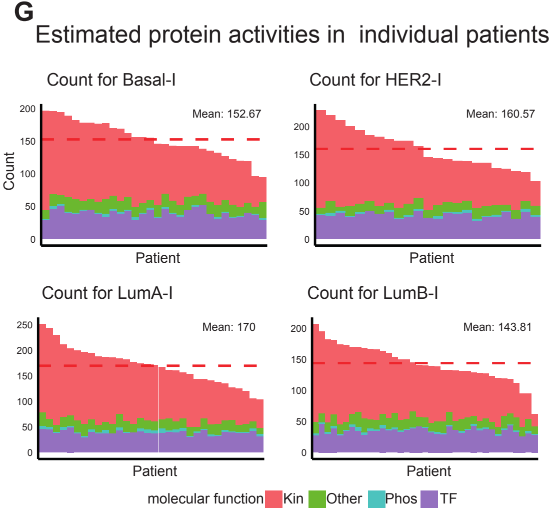
G
Estimated protein activities in individual patients
Count for Basal-I
Mean: 152.67
Count for HER2-I
Mean: 160.57
Count for LumA-I
Mean: 170
Count for LumB-I
Mean: 143.81
Count
Patient
molecular function
Kin
Other
Phos
TF
