# Supplementary material for: PatientProfiler: building patient-specific signaling models from proteogenomic data
Source: Mol Syst Biol. 2025 Oct 10;21(12):1845–65. doi: 10.1038/s44320-025-00160-y (PMC12672659; doi:10.1038/s44320-025-00160-y)
Supplement: Supplementary file 7 — Source data Fig. 2 [file 44320_2025_160_MOESM7_ESM.zip › Figure 2/2F/2F.pdf]

F

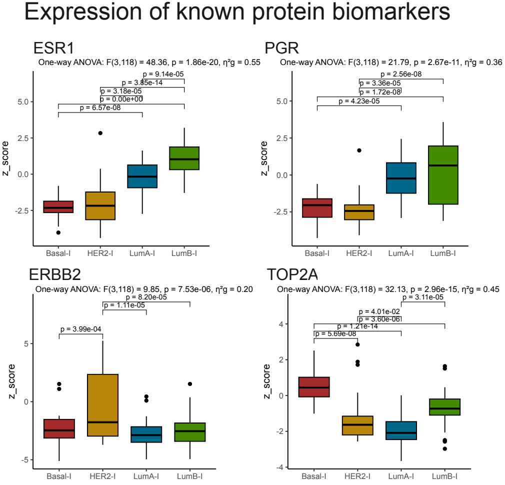
Expression of known protein biomarkers
ESR1
One-way ANOVA: F(3,118) = 48.36, p = 1.86e-20, η²g = 0.55
p = 9.14e-05
p = 3.85e-14
p = 3.18e-05
p = 0.00e+00
p = 6.57e-08
PGR
One-way ANOVA: F(3,118) = 21.79, p = 2.67e-11, η²g = 0.36
p = 2.56e-08
p = 3.36e-05
p = 1.72e-08
p = 4.23e-05
ERBB2
One-way ANOVA: F(3,118) = 9.85, p = 7.53e-06, η²g = 0.20
p = 8.20e-05
p = 1.11e-05
p = 3.99e-04
TOP2A
One-way ANOVA: F(3,118) = 32.13, p = 2.96e-15, η²g = 0.45
p = 3.11e-05
p = 4.01e-02
p = 3.60e-06
p = 1.21e-14
p = 5.69e-08
z_score
Basal-I
HER2-I
LumA-I
LumB-I
