# Supplementary material for: PatientProfiler: building patient-specific signaling models from proteogenomic data
Source: Mol Syst Biol. 2025 Oct 10;21(12):1845–65. doi: 10.1038/s44320-025-00160-y (PMC12672659; doi:10.1038/s44320-025-00160-y)
Supplement: Supplementary file 7 — Source data Fig. 2 [file 44320_2025_160_MOESM7_ESM.zip › Figure 2/2C/2C.pdf]

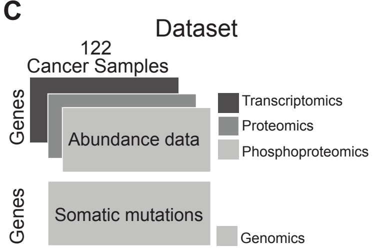
C
Dataset
122
Cancer Samples
Genes
Abundance data
Transcriptomics
Proteomics
Phosphoproteomics
Genes
Somatic mutations
Genomics
