# Supplementary material for: PatientProfiler: building patient-specific signaling models from proteogenomic data
Source: Mol Syst Biol. 2025 Oct 10;21(12):1845–65. doi: 10.1038/s44320-025-00160-y (PMC12672659; doi:10.1038/s44320-025-00160-y)
Supplement: Supplementary file 7 — Source data Fig. 2 [file 44320_2025_160_MOESM7_ESM.zip › Figure 2/2B/2B.pdf]

B

	Receptor			Proliferative index
Subtype	ESR1	PGR	ERBB2	TOP2A
Basal-I	-	-	-	high
Her2-I	-	-	+	
LumA-I	+	+	-	low
LumB-I	+	+/-	-	high
