# Supplementary figures and images for: PatientProfiler: building patient-specific signaling models from proteogenomic data
Source: Mol Syst Biol. 2025 Oct 10;21(12):1845–65. doi: 10.1038/s44320-025-00160-y (PMC12672659; doi:10.1038/s44320-025-00160-y)

# A The patient-specific “cell reprogramming” in Breast cancer

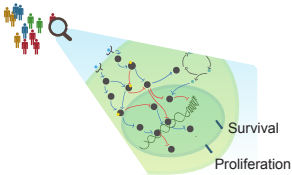

Supplement: Supplementary file 7 — Source data Fig. 2 [file 44320_2025_160_MOESM7_ESM.zip › Figure 2/2A/2A.pdf]

**D**

-omic dataset size

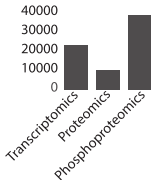

Supplement: Supplementary file 7 — Source data Fig. 2 [file 44320_2025_160_MOESM7_ESM.zip › Figure 2/2D/2D.pdf]

E

## Subtypes

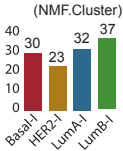

Supplement: Supplementary file 7 — Source data Fig. 2 [file 44320_2025_160_MOESM7_ESM.zip › Figure 2/2E/2E.pdf]

C

## Phenotype activity status

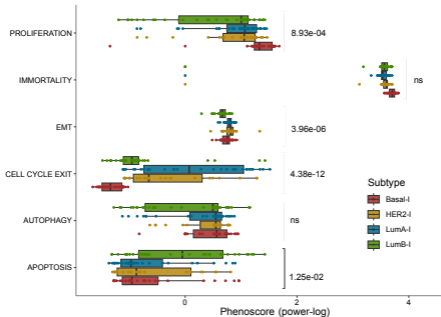

Supplement: Supplementary file 8 — Source data Fig. 3 [file 44320_2025_160_MOESM8_ESM.zip › Figure 3/3C/3C.pdf]

Functional circuit of patient X01BR010, HER2-I

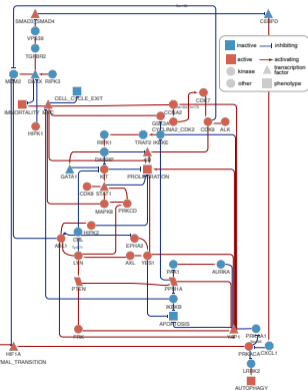

Supplement: Supplementary file 8 — Source data Fig. 3 [file 44320_2025_160_MOESM8_ESM.zip › Figure 3/3A/3A.pdf]

## Communities subdivision

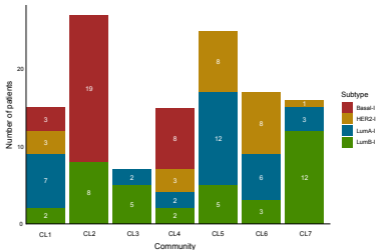

Supplement: Supplementary file 9 — Source data Fig. 4 [file 44320_2025_160_MOESM9_ESM.zip › Figure 4/4B/4B.pdf]

## Phenotype activity status

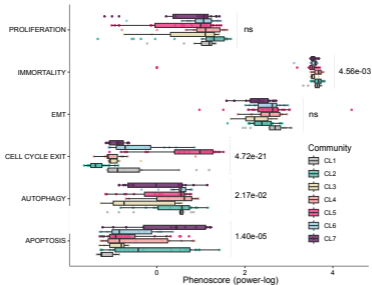

Supplement: Supplementary file 9 — Source data Fig. 4 [file 44320_2025_160_MOESM9_ESM.zip › Figure 4/4C/4C.pdf]

## Strategy description

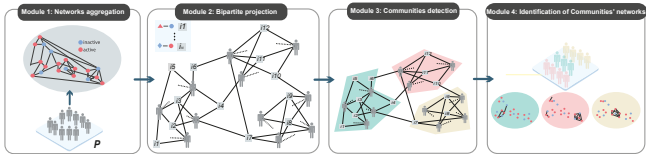

Supplement: Supplementary file 9 — Source data Fig. 4 [file 44320_2025_160_MOESM9_ESM.zip › Figure 4/4A/4A.pdf]

# Protein levels of CL2 and CL4 nodes in TCGA patients

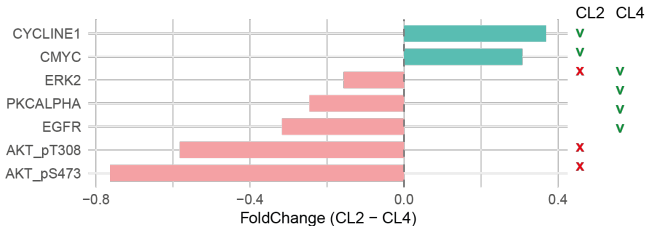

Signature2-enriched      Signature4-enriched

Supplement: Supplementary file 10 — Source data Fig. 5 [file 44320_2025_160_MOESM10_ESM.zip › Figure 5/5C/5C.pdf]

**A**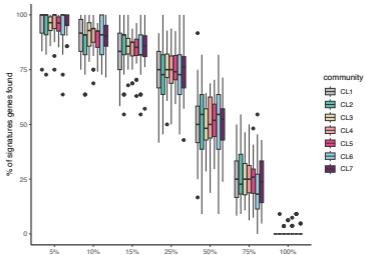

Supplement: Supplementary file 11 — Source data Fig. 6 [file 44320_2025_160_MOESM11_ESM.zip › Figure 6/6A/6A.pdf]

**B**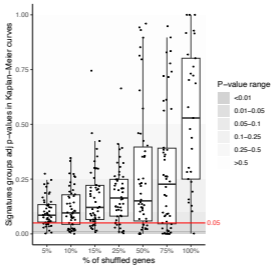

Supplement: Supplementary file 11 — Source data Fig. 6 [file 44320_2025_160_MOESM11_ESM.zip › Figure 6/6B/6B.pdf]
