# Supplementary material for: PatientProfiler: building patient-specific signaling models from proteogenomic data
Source: Mol Syst Biol. 2025 Oct 10;21(12):1845–65. doi: 10.1038/s44320-025-00160-y (PMC12672659; doi:10.1038/s44320-025-00160-y)

B

Network size in individual patients - Full Interactome

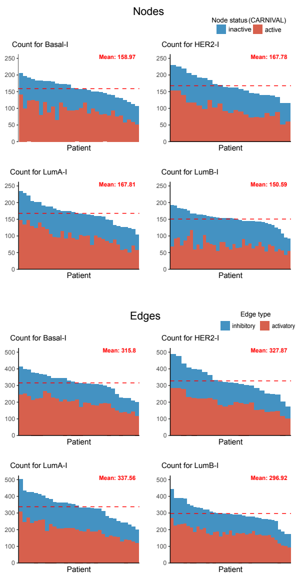

Supplement: Supplementary file 8 — Source data Fig. 3 [file 44320_2025_160_MOESM8_ESM.zip › Figure 3/3B/3B.pdf]
