# Supplementary material for: PatientProfiler: building patient-specific signaling models from proteogenomic data
Source: Mol Syst Biol. 2025 Oct 10;21(12):1845–65. doi: 10.1038/s44320-025-00160-y (PMC12672659; doi:10.1038/s44320-025-00160-y)

A

Circuits driving apoptosis inhibition and proliferation activation

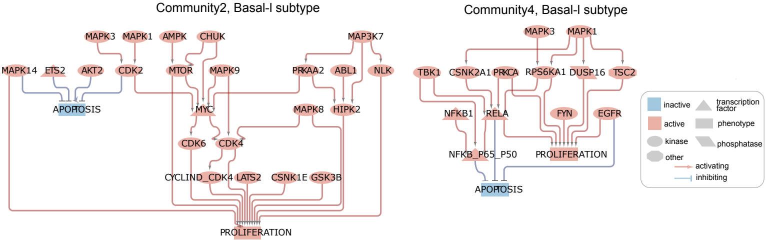

Supplement: Supplementary file 10 — Source data Fig. 5 [file 44320_2025_160_MOESM10_ESM.zip › Figure 5/5A/5A.pdf]
