# Supplementary material for: PatientProfiler: building patient-specific signaling models from proteogenomic data
Source: Mol Syst Biol. 2025 Oct 10;21(12):1845–65. doi: 10.1038/s44320-025-00160-y (PMC12672659; doi:10.1038/s44320-025-00160-y)
Supplement: Supplementary file 10 — Source data Fig. 5 [file 44320_2025_160_MOESM10_ESM.zip › Figure 5/5B/5B.pdf]

Survival analysis within TCGA

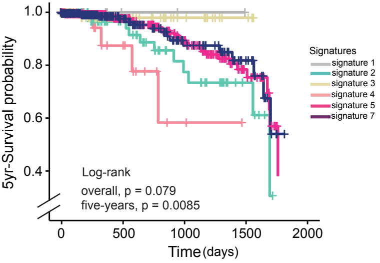

Number at risk

Signatures \ Time (days)	0	500	1000	1500	2000
signature 1	5	2	1	0	0
signature 2	85	38	19	6	0
signature 3	71	28	13	4	0
signature 4	24	11	2	0	0
signature 5	438	176	96	26	0
signature 7	281	98	45	20	0
